# Recent Advances in Developing Insect Natural Products as Potential Modern Day Medicines

**DOI:** 10.1155/2014/904958

**Published:** 2014-05-06

**Authors:** Norman Ratcliffe, Patricia Azambuja, Cicero Brasileiro Mello

**Affiliations:** ^1^Laboratório de Biologia de Insetos, Departamento de Biologia Geral, Universidade Federal Fluminense, Niterói, RJ, Brazil; ^2^Department of Biosciences, College of Science, Swansea University, Singleton Park, Swansea SA2 8PP, UK; ^3^Laboratório de Bioquímica e Fisiologia de Insetos, Instituto Oswaldo Cruz, Fundação Oswaldo Cruz, Avenida Brasil 4365, 21045-900 Rio de Janeiro, RJ, Brazil

## Abstract

Except for honey as food, and silk for clothing and pollination of plants, people give little thought to the benefits of insects in their lives. This overview briefly describes significant recent advances in developing insect natural products as potential new medicinal drugs. This is an exciting and rapidly expanding new field since insects are hugely variable and have utilised an enormous range of natural products to survive environmental perturbations for 100s of millions of years. There is thus a treasure chest of untapped resources waiting to be discovered. Insects products, such as silk and honey, have already been utilised for thousands of years, and extracts of insects have been produced for use in Folk Medicine around the world, but only with the development of modern molecular and biochemical techniques has it become feasible to manipulate and bioengineer insect natural products into modern medicines. Utilising knowledge gleaned from Insect Folk Medicines, this review describes modern research into bioengineering honey and venom from bees, silk, cantharidin, antimicrobial peptides, and maggot secretions and anticoagulants from blood-sucking insects into medicines. Problems and solutions encountered in these endeavours are described and indicate that the future is bright for new insect derived pharmaceuticals treatments and medicines.

## 1. Introduction

Previously, a number of overviews on insect natural products and their potential for development into drugs to treat human diseases have been published [1–3]. Recently, however, there have been additional advances in this field. The present review therefore focuses on these as well as their implication for studying mammalian physiology and the immune reactions to human pathogens.

Surprisingly, despite the success of insects in terms of numbers and diversity, the most successful drugs derived from natural products, including artemisinin, quinine, aspirin, cocodamol, simvastatin, and cyclosporine, have been isolated from plants, marine organisms, and microbes [3, 4]. Altogether, 939 nature-derived approved drugs were developed between 1961 and 2010 [4] but none of these were from insects and only a few originated from invertebrates such as leeches, sponges, and cone snails. Difficulties in species identification, drug toxicity, development costs, and large scale production [3] partially explain the reason for the slow progress in developing insect products as potential modern medicines. However, since modern genomics,* in silico* drug design and high throughput screening have failed to yield new generations of novel drugs; there is now renewed interest in more traditional methods of screening using the huge diversity of animals, plants, and microbes available [5]. Furthermore, more traditional biochemical screening techniques have now resulted in notable progress in developing therapeutics from arthropods, including melittin from bees [6], alloferon from blowflies [7], and anticoagulants from ticks [8].

## 2. Use of Insects in Folk Medicine

Despite the fact that insects have not been a rich source of modern drugs, they have, for thousands of years, provided many invaluable natural substances, including silk and honey products (royal jelly, beeswax, pollen, and propolis). Insect secretions and ground-up bodies have commonly been used in Folklore Medicine not only in China and Bahia but also in India, Asia, Africa, and Mexico (e.g., [2, 9, 10]). Unfortunately, two of the most fascinating accounts of the use of insects in Folk Medicine have not been published in mainline scientific journals but are well worth reading [11, 12]. One of these includes an unpublished book by Lockhart [11], while the other is a blog describing the experiences of the author with the use of ants in the Bolivian Amazon [12]. A more recent published review on insects as medicines deserves mentioning too as it presents alluring accounts of Insect Folk Medicine in India and Zaire as well as the use of insects as food [13].

Insects and insect extracts have been used in Folk Medicine for a huge range of conditions including arthritis treatment with* Pseudomyrmex* ant venom which resulted in US patent number 4, 247, 540 in 1981 [11, 12]. Amazonian Indians also diagnosed diabetes by watching to see whether ants swarmed over urine which in diabetics contains high levels of sugar to attract the ants [11]. Particularly thought provoking is the account of ants being used to cure lethargy [11]. Altogether in China, 1,700 medicines have been produced from* ca.* 300 insect species while 42 species have been used as Folk Medicines in Bahia [14].

Only a few Insect Folk Medicines have undergone exhaustive clinical trials to prove their efficacy. Scientists, however, are now using knowledge accrued from exponents of Folk Medicine to develop potential new medicines for treating intractable diseases such as cancer and the problems associated with newly emerging antibiotic-resistant bacteria. In the following examples of the development of insect products as potential modern medicines, there is already a long history of the use of these substances in Folk Medicine.

## 3. Bee, Wasp, and Ant Products

**Figure 1:**
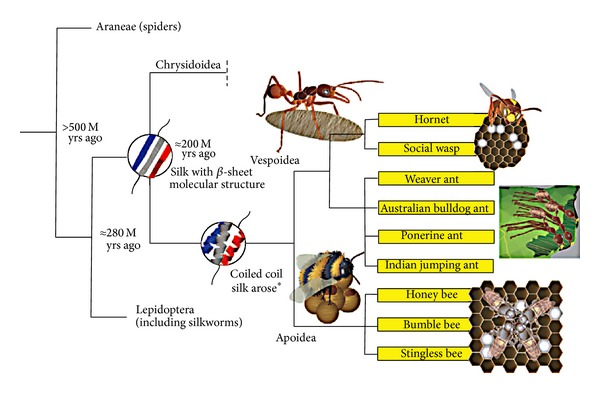
Schematic showing approximate evolutionary relationship of spiders, silkworms, bees, wasps, and ants mentioned in this review. Figure from Tara D. Sutherland, Sarah Weisman, Andrew A. Walker, and Stephen T. Mudie, “The Coiled Coil Silk of Bees, Ants, and Hornets,”* Biopolymers* volume 97, Issue 6, pp. 446–454, 2012 (DOI 10.1002/bip.21702) published by John Wiley and Sons with permission.

Bee, wasp, and ant products, including honey and venom, have been used in Folk Medicine for thousands of years for treating wounds, ulcers, inflammation, infections, pain, cancer, and allergies [3]. Studies of natural products from hymenopterans (Figure 1) have mainly concentrated on honey bee compounds because of the ready availability of large numbers of these insects reared under relatively constant controlled conditions.

### 3.1. Honey Products from Bees

Recently, the use of honey for wound healing has been extensively reviewed [15–17]. These studies demonstrated the efficacy of honey in wound repair and sterilization of infected wounds and generally supported the use of honey in clinical practice, but only with certain types of wounds and after additional clinical trials [15]. The evidence available, for example, includes 19 randomized controlled trials with 2554 patients which suggested that honey improved healing times but only in mild-to-moderate superficial burns and not in full thickness burns [18]. In addition, more recently, an analysis of 44 Cochrane reviews also provided robust evidence that in some wound care interventions topical honey application reduced healing times of burns [19].

Honey is a complex mixture of substances and progress is being made at the molecular level in understanding the functions of the various components on cells and the effectiveness of honey in treating a range of human ailments. For example, Tonks et al. [21] isolated a 5.8 kDa honey component which stimulated the production of the TNF-alpha cytokine via TLR4 in human monocyte cultures. TNF-alpha is involved in the repair and regeneration of tissues.

The antimicrobial activity of honey is probably due to a combination of low pH, high osmolality, and hydrogen peroxide generation together with defensin-1 and methylglyoxal, with the latter an aldehyde generated from pyruvic acid [3, 22]. Interestingly, Kwakman et al. [22] recently showed that Revamil and Manuka honeys have different antibacterial components, with the former containing defensin-1, hydrogen peroxide, and methylglyoxal, while the latter only had methylglyoxal at 44 times the concentration of Revamil. In addition, Manuka honey was also shown to contain other unidentified antibacterial factors. Great variations in antimicrobial properties have also been discovered for a range of honeys, limiting those suitable for use in medicine [23].

There is great recent interest in the antimicrobial activity of honey against important antibiotic-resistant human pathogens (reviewed in [17]). These studies showed, for example, inhibition of Gram-positive MRSA (methicillin resistant* Staphylococcus aureus*), of vancomycin-sensitive and resistant* Enterococci *(VSE and VRE, e.g., [24]), and of* Streptococcus *species isolated from wounds [25]. Honey also impacts Gram-negative bacteria associated with wounds such as* Pseudomonas aeruginosa, Stenotrophomonas *species, and* Acinetobacter baumannii* (e.g., [17]). Manuka honey appears to inhibit cell division in MRSA [26], while, with* P. aeruginosa,* the cell wall is destabilised and lysis occurs [27]. Bacterial DNA degradation in pathogens has also been reported with Buckwheat honey [28]. Finally, honey can not only inhibits planktonic bacteria but also prevents the formation of biofilms [17, 29] that form, for example, on surgical implants, thus causing prosthesis failure and additional patient distress. A review has been published of recent patents resulting from all this work on antibiotics from hives [30].

The above benefits of honey in wound healing and bacterial inhibition have resulted in the development of special dressings to treat different types of wounds. Some of these are in the form of ointments or gels, while others are actual dressings made from mixes of alginate with honey [17].

Other honey products have also been shown to have antimicrobial activity so that propolis and the apalbumins in royal jelly have been reported to inhibit bacteria [3, 31]. Propolis also has a synergistic effect with antimicrobial drugs in the treatment of experimental* S. aureus* keratitis and diminishes the resistance of the bacterial cell walls to antibiotics (reviewed in [31]). The effect of propolis on oral* Streptococcus mutans *also indicates the possible development of this factor as a cariostatic agent to control caries and other infectious diseases of the mouth [31].

Regarding the anticancer properties of honey products, these have been reported previously with a fatty acid in royal jelly and the flavonoids in propolis responsible [3]. An excellent overview of the immunomodulatory and antitumour activity of bee honey in experimental and clinical studies was published in 2009 [32]. Further recent progress has been made in understanding more details of the anticancer properties of the mixture of polyphenols present in honey, propolis, and royal jelly [20]. An indication of the complexity of the phenolic mixture in honey is given in Table 1.

Of these compounds, quercetin has been shown to enhance the apoptotic ability of anti-CD95 and rTRAIL (recombinant tumor necrosis factor-related apoptosis inducing ligand) in acute lymphocytic leukemia [33]. In addition, details of the ability of polyphenols isolated from propolis to overcome the resistance of cancer cells to TRAIL-mediated apoptosis have recently been reviewed [34]. The possible use of propolis as a dietary supplement in a cancer preventative strategy was emphasized [34].

Other phenolic compounds in honey in Table 1, with anticancer properties, include apigenin and acacetin which not only induce caspase-dependent apoptosis in human leukemia cells* in vitro* but the former also produced apoptosis-mediated inhibition of U937 leukemic cell xenografts in mice [35]. Other phenolic compounds in Table 1 also have antileukemic cell growth inhibition* in vitro* mediated by apoptosis [20]. None of these researches has, to date, led to new chemotherapeutic agents but the information from* in vitro* studies on human cancer cells should provide clues to help the future development of new medicines [20].

More promising, for the more immediate development of new anticancer therapeutics from honey products, is the work of Fernandez-Cabezudo et al. [36]. Initially, they confirmed the killing properties of Manuka honey on three cancer cell lines via a caspase 9-dependent apoptotic pathway inducing caspase 3, reducing Bcl-2 expression, and leading to DNA fragmentation and cell death. Subsequently, they injected Manuka honey alone or in combination with a chemotherapeutic agent (taxol) into mice implanted with syngeneic melanoma cells and recorded inhibition of tumour growth and host survival. Controls injected solely with Manuka honey showed 33% inhibition of tumour growth. The combination group of Manuka honey plus taxol showed no increase in tumour inhibition in comparison with the taxol group alone; however, what was remarkable was the highly significant improvement in survival of mice in the combination group. This study indicates the potential of Manuka honey in alleviating chemotherapeutic toxicity [36] and improving patient survival.

### 3.2. Bee, Wasp, and Ant Venoms

Bee venom therapy has been used in Folk Medicine for many thousands of years for treating a range of ailments from arthritis, rheumatism, skin diseases, multiple sclerosis, cancer, infections, and pain (reviewed in [1, 3]). Apart from bee venom, the venoms of many other stinging insects, such as wasps and ants, contain a large range of practically unexplored compounds awaiting discovery and development into the medicines of tomorrow. For example, some ant and parasitoid wasp venoms may contain 75 or more different components [37, 38].

Although bee venom therapy has been widely used, it has neither, as yet, been approved by drug safety authorities nor commonly adopted by conventional medicine. However, there are some crude formulations available, including Apiven in France, produced from the crude venom of honey bees [39].

Honey bee venom is a mixture of at least 20 compounds, including 1/. active peptides such as melittin, apamine, mast cell degranulating peptide (MCD), and adolapin, 2/. the enzymes phospholipase A_2_ (PLA_2_), and hyaluronidase, and 3/. the active amines, histamine, serotonin, and catecholamine. Of these components, melittin and phospholipase A_2_ make up 40–60% and 10–12% dry weight of venom, respectively [3, 40].

Despite the multifunctional use of honey bee venom in Folk Medicine, recent research has focused mainly on melittin and its anticancer properties, although apamine and phospholipase A_2_ have also received some attention [3, 40]. There is an extensive literature on melittin which probably reflects the great potential of this peptide for development as a therapeutic medicine for treating different types of cancers. Melittin is a water soluble molecule, with cationic and amphipathic properties which enhance the electrostatic binding to the anionic cell membranes of many bacteria and cancer cells. Melittin contains 26 amino acids (GIGAVLKVLTTGLPALISWIKRKRQQ-NH2) which in the venom reservoir have a tetrameric structure (reviewed in [3, 41]). Upon binding, melittin induces cytolysis of most membranes such as those of normal mammalian cells. Thus, melittin is cytotoxic* in vivo* which has hindered its therapeutic development, despite the fact that it inhibits or kills a range of cancer cell types, such as melanoma, osteosarcoma, leukemic, ovarian, prostate, hepatic, renal, bladder, and mammary gland cells [42].

The precise mode of action of melittin in killing cancer cells is not fully understood although recent advances in understanding its cytolytic effect have been made [43]. At low concentrations, melittin induces transient pore formation in the cell membrane due to tension resulting from one-sided binding of melittin to the outer cell membrane leaflet. At higher concentrations, melittin binding results in the formation of stable pores in the cell membrane leading to cell lysis as the melittin concentration increases and the pores coalesces. Melittin has multiple effects on cells (reviewed in [42]). These effects range from hormone induction, membrane protein aggregation, and changes in membrane potential to stimulation of G-protein enzymes and PLA_2_, as well as a role in cell signal induction [42]. The possible effects of melittin and other bee venom components on cancer cells and host immunity involve inhibition of calmodulin and  NF-*κ*B. These effects, in turn, would inhibit cancer cell proliferation, invasion and metastasis, and angiogenesis and induce apoptosis [42].

It appears that bee venom induces apoptosis, necrosis, and lysis of tumour cells and, at the same time, can produce immunosuppressive and/or immunostimulation in the host [42]. Melittin apparently induces apoptosis via activation of the PLA_2_ in cancer cells, especially those transformed by the ras oncogene [44]. Excellent detailed reviews of the interaction of melittin and other bee components with tumour cells and the therapeutic potential of bee venom have been published by Gajski and Garaj-Vrhovac [40] and Oršolić [42].

**Figure 2:**
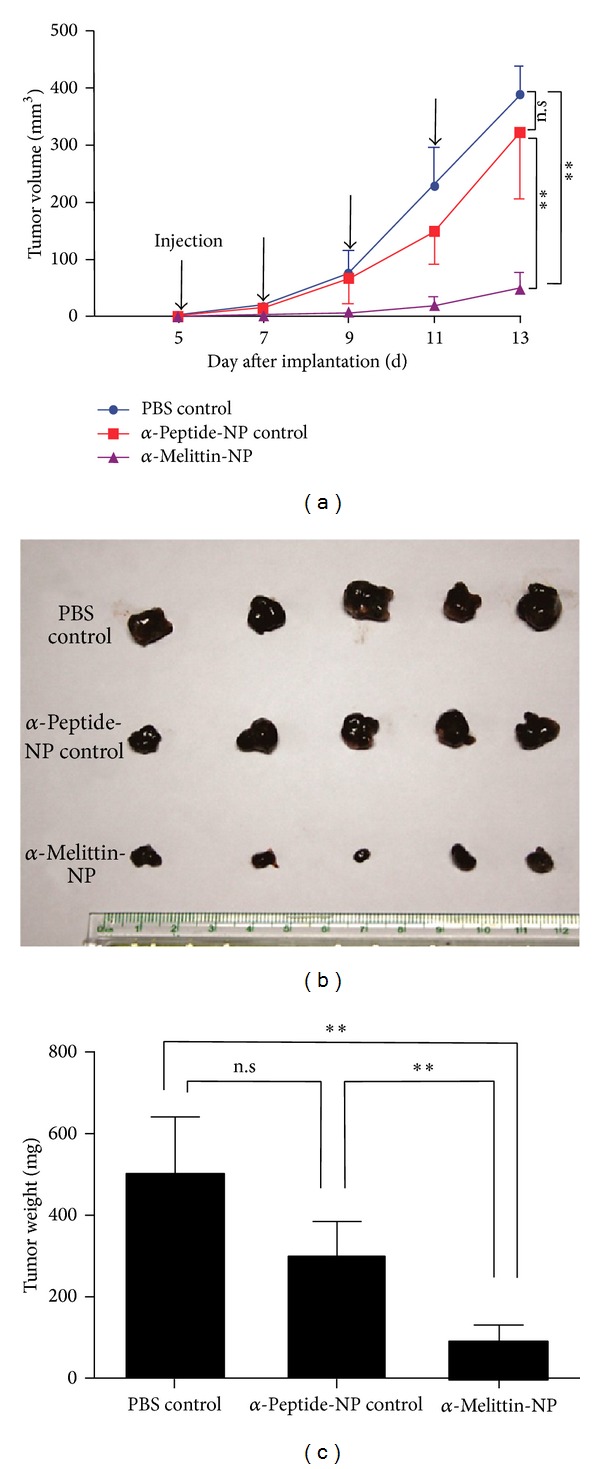
Evaluation* in vivo* of the effect of *α*-melittin nanoparticles on the inhibition of melanoma development. (a) Tumor volume over time showing that only the *α*-melittin nanoparticle group was significantly inhibited. (b) Comparative sizes and (c) volumes of the excised tumors between different groups after 13 days growth. Means ± SD, *n* = 5, **P < 0.01. Reprinted with permission from C. Huang, H. Jin, and Y. Qian et al., “Hybrid melittin cytolytic peptide-driven ultrasmall lipid nanoparticles block melanoma growth* in vivo*,”* ACS Nano*, volume 7, number 7, 5791-5800, 2013. (DOI: 10.1021/nn400683s). Copyright (2013) American Chemical Society.

In efforts to overcome the cytolytic properties of melittin and to harness its anticancer properties, scientists have adopted several strategies (Table 2). First, since cancer cells have higher anionic surface charges and are more sensitive to melittin than normal cells then melittin can be diluted to levels able to kill lung cancer cells* in vitro,* while normal cells are unaffected [45]. Second, Zhao et al. [46] modified the melittin chain by mutating Val 5 to Arg, Ala 15 to Arg and deleting Leu 15 which significantly reduces the haemolytic properties but maintains its inhibitory effects. Third, an alternative strategy involves using a synthetic melittin peptide coupled to a delivery vehicle such as the beta chain of human chorionic gonadotropin (hecate-CGb). Cells with upregulated expression of hormone receptors, such as ovarian, testicular, and adrenocortical tumours in mice, can then be specifically targeted* in vivo* (e.g., [47]). Fourth, is similar to three (above) but uses melittin linked to a specific homing peptide for hepatocellular carcinoma cells* in vitro* [48]. The importance of this study is that it identified a specific homing peptide for the cancer cells using a phage display technique for screening and identification of the novel peptide. Fifth, by using gene therapy in which expression constructs carry the gene for melittin into tumours and induce antitumour effects and increased tumour latency [49]. Many of the previous bioconjugate techniques, however, still induce some haemolysis of normal cells. The sixth, and final strategy, is probably the most promising for the therapeutic use of melittin. It involves using nanoparticles to deliver melittin specifically to kill melanomas and other cancers* in vivo* with no cytotoxicity towards normal cells [50]. The nanoparticles were targeted to the tumours by incorporating an avb3 integrin-binding ligand [3, 50]. The resultant reduction of the tumour load in the experimental mice was quite startling. This technology has been taken a step further since the nanoparticles used in the Soman et al. [50] study were quite large (ca. 270 nm) and probably failed to penetrate solid tumours efficiently [51]. Thus, Huang et al. [51] designed an ultrasmall, neutral charged, lipid nanoparticle (ca. 20 nm) containing a hybrid *α*-melittin which inhibited the growth of the melanoma cells in mice* in vivo* by 82.3% compared with the PBS controls (Figure 2).

Apart from the role of melittin in killing cancer cells, PLA_2_ and apamine in bee venom also have anticancer activities. For example, venom PLA_2_ acting synergistically with the cell membrane phospholipid, phosphatidylinositol-(3,4)-bisphosphate, has been shown to be involved in inhibition of tumour cell growth and potent cell lysis (detailed in [42]). Apamine too could potentially be developed as an anticancer therapeutic agent since it reactivates the p53 tumour suppressor pathway and would trigger the rapid elimination of tumours (reviewed in [42]).

Finally, the antimicrobial properties of melittin are well known and activity* in vitro* has been recorded against a range of microbes including not only* Escherichia coli* and* Staphylococcus aureus* but also* Borrelia burgdorferi* and* Candida albicans* [3]. Again, the cytolytic activity of this molecule for mammalian cells has been a barrier to its development as a therapeutic drug [3]. The insect antimicrobial peptides are discussed later in this review (see Section 6, “Antimicrobial Peptides” (AMPs)). Recent developments in the use of melittin as an AMP have reported a synergistic effect when melittin was combined with antibiotics against Gram-positive bacteria even at concentrations as low as 0.5× MIC [52]. In addition, melittin loaded nanoparticle constructs have been shown to inhibit HIV-1 infectivity of TZM-bl reporter cells (a strain of HaLa cells) but, at the same time, to be nontoxic to these and to VK2 vaginal epithelial cells. Thus, melittin nanoparticle constructs have the potential to be developed for use as topical therapeutic vaginal virucides [53].

## 4. Silk

Silk has been produced for at least 5,000 years with nearly 75% now originating from China [3]. In Chinese medicine, silk has been used for a variety of human conditions including the relief of spasms and flatulence. Interestingly, silkworm larvae have also been prescribed for treating impotence [54] only for, subsequently, a vasodilator compound enhancing NO production to be extracted from* Bombyx mori* larvae and to be a candidate for the therapeutic treatment of vascular impotency [54].

Interest in the medical or industrial use of silk is not confined to the silk produced by silkworms since many other insects such as the Hymenoptera (bees, wasps, hornets, and ants) and the Trichoptera (caddis flies) [55], as well as the Arachnida (spiders) [56], produce silk. The macromolecular structure of the silks from different arthropods varies according to their function in the life of the animal. Basically, the main structure of silkworm silk consists of fibroin protein fibres held together by a sticky protein called serecin. Boiling* B. mori* cocoons remove the serecin glue to release the fibroin fibres for subsequent processing (3). In the larvae of bees, ants, and hornets, the silk produced has a coiled coil molecular structure, in contrast to other hymenopterans, as well as spider draglines (safety lines) and* B. mori* cocoons, in which the silk proteins form extended ß-sheets [57]. The coiled coil silk proteins are small and ideal as structural materials to strengthen the walls of the brood comb cells. The silk also absorbs water and maintains the high humidity and constant temperature necessary for pupal development [57].

Silk is not prescribed in modern medicine; however, it was used previously for medical sutures but now has been replaced by synthetic polymers. The ingenuity of science continues to amaze with silk recently produced as biomaterials for the transport and delivery of dugs around the human body [58, 59] and for tissue engineering [60]. This progress in the use of silk resulted from the publication of the* B. mori* genome in 2008 [61] which led to gene cloning and modification to allow the expression of silk in a variety of vectors. It was then possible to produce synthetic silk in different conformations, such as scaffolds, films, and nanoparticles, for use in medicine [60].

What are the properties of silk that make it so attractive for use in medicine and which have fuelled recent intensive study? Silk is slow to biodegrade and biocompatible with the human body, although inflammatory responses have been recorded [56]. In addition, silk has good self-assembly properties and high tensile strength with manipulatable structure and composition [62]. Finally, silk can be produced in aqueous solutions in order to avoid inactivation of the associated drug or gene and the rate of delivery of which can be modulated by controlling the speed of degradation of the silk vehicle [59].

Scientists have been developing both kinds of spider and silkworm silk for potential uses in medicine. Silkworms silk is available in large quantities without recombinant methods necessary. However, interest persists in spider silk, despite the fact that it is impossible to develop large-scale farming of spiders, due to the fact that spider silk is extremely strong, flexible, and tough and therefore particularly promising for the production of biomaterials. The toughness of silk is due to the presence of numerous interlocking poly-alanine and glycine-alanine subunits which strengthen the silk proteins [63]. However, the spider silk proteins are long and this has caused problems in recombinant technology due to, for example, the repetitive sequences inducing genetic instability [56]. Some of these problems with spider silk have, however, been resolved, by various strategies. Thus, recombinant technology has been used to produce spider silk in* E. coli*, yeast, plants, and mammalian cells, as well as in the milk of mice and goats, all of which present unique problems in execution [56]. One study even reports the use of piggyBac vectors to create transgenic silkworms producing chimera silkworm/spider silk proteins in which the composite fibres are as tough as native spider dragline silk [64].

The recent work of Numata et al. [58, 59, 62, 65–67] indicates that rapid progress is being made in the development of silk for use in medicine (Table 3). They have used recombinant synthesis of spider silk in* E. coli *to produce silk polymers which were then used for the production of microspheres/nanoparticles and block copolymers for the targeted delivery of drugs to cancer cells or to act as gene vectors [58, 59]. For example, nanoparticles enclosing curcumin have been shown to be promising for treating breast cancer [59]. The block copolymers are engineered containing silk with polylysine, for example, and cell-binding motifs such as RGD for targeting cells together with a therapeutic drug. A variation of the copolymer is to include plasmid DNA for transfecting target cells with specific genes [58, 59]. More recently, further improvements have been made in the specificity of the silk polymer delivery system by introducing cationic motifs and tumour specific homing peptides and reducing the size of the silk carrier and the pDNA [65–67].

There are also numerous recent studies of the use of silk in tissue engineering with an enormously active group based in the Department of Biomedical Engineering at Tufts University working on both spider and silkworm silks. The work under Drs. Kaplan and Omenetto has looked at the use of silk polymers for tissue engineering, vaccine production without the need for refrigerated storage, and cosmetic surgery. A number of start-up companies have been spawned and the future prospects have great potential (see http://www.techtransfer.tufts.edu/tufts-silk-portfolio/). Recent research from this group has reviewed the strategies to produce spider silk by recombinant DNA [75]. In addition, they have looked at silk-heparin biomaterials for vascular tissue engineering [69], silk hydrogels for treating breast cancer [70], antibiotic-releasing silk biomaterials for infections [71], electrical stimulation of silk films for enhancement of neural growth and silk containing dressings for increased wound healing [72], and silk protein matrices which thermostabilize labile vaccines and antibiotics [73]. The latter development is very exciting and could potentially solve the problem of transporting vaccines to remote parts of Africa when vaccines against malaria are finally produced (Table 3). In many of these studies, growth stimulating factors or drugs are incorporated into the polymers and slowly released into the target tissues [69–71].

Finally, many other studies have described the potential use of silk polymers in medicine [74, 76]. For example, Sheng et al. [74] using vitamin-E loaded silk nanofibrous mats showed enhancement of skin fibroblast growth, and therefore this technique can be developed for skin regeneration in the future (Table 3). The FDA approval of silkworm silk for use in the human body has no doubt stimulated interest in this exciting research area.

## 5. Cantharidin from Blister Beetles and Other Small Molecules

Blister beetles belong to the Coleopteran Family Meloidae which contains* ca. *2500 species [77]. Many of these insects produce toxic defensive secretions which upon contact with the skin cause blistering. One such toxin is cantharidin which has been extracted from* Mylabris caragnae, *the dried bodies of which have been used in Chinese Folk Medicine since the 13th century for the removal of warts [78] and for over 2000 years for the treatment of cancer. Other uses include the treatment of rabies and impotence although it is highly toxic affecting the gut and kidneys [3, 78]. The fatal dose, causing renal failure, is between 10 and 65 mg and this toxicity has hindered cantharidin development as an anticancer drug [78]. In addition, the dried bodies of another beetle,* Lytta vesicatoria,* supposedly have aphrodisiac properties and were sold as a powder called “Spanish Fly” [3]. In fact, the male beetle produces cantharidin and offers it to the female as a precopulatory incentive and she uses it to protect her eggs.

There is increasing interest in the use of cantharidin and its derivatives for the treatment of a range of cancers including hepatic, colorectal, bladder, breast, melanomas, pancreatic, and leukemia [3]. The anticancer properties of cantharidin result in arrest of the cell cycle in G2/M phase, apoptosis, and oxygen radical damage to DNA [79]. However, the potential of this small molecule and its derivatives in medicine is not confined to their anticancer properties as they have also been reported to have activity against parasites such as* Plasmodium falciparum* and* Leishmania major* [80, 81].

Cantharidin is a monoterpene (exo,exo-2,3-dimethyl-7-oxabicyclo[2.2.1]heptane-2,3-dicarboxylic acid anhydride), stored in the beetle haemolymph and making up about 5% of body dry weight [78]. Organic chemists have been working to produce derivatives which are bioactive but less toxic. In consequence, the norcantharidins have been produced with anticancer activity but reduced toxicity [78]. In addition, a new class of anticancer compounds, the cantharimides, has been discovered from a Chinese blister beetle,* Mylabris phalerate*, closely related to cantharidin but with improved water solubility and toxicity against human hepatocellular carcinoma cell lines [82]. An excellent account of the strategies adopted to produce improved cantharidin and cantharimide analogues is given in a review by Galvis et al. [78] and many of the derivatives described have higher bioactivity and less toxicity.

Despite the development of less toxic analogues, there is still concern about the use of cantharidin in the clinical situation with trials mainly limited to external use on warts [3]. However, scientists have continued their research and now much more is known about the mode of action of cantharidin so that new strategies for drug administration are being developed. A recent limited clinical trial involving combining cantharidin with chemotherapy for the treatment of gastric cancer has been completed. The results showed the beneficial effects of the cantharidin by a reduction of the serious side effects usually associated with chemotherapy for gastric cancer [83].

Research has also shown that cantharidin is an inhibitor of phosphoprotein phosphatases 1 (PP1) and 2A (PP2A) which results in DNA damage and apoptosis [78, 84]. These enzymes are involved in regulation of metabolism and the initiation of signal transduction in cells resulting in cell division. Thus, cantharidin may represent a small molecule able to switch cancer cells division and carcinogenesis off/on as well as to probe the key regulatory role of PPA2 in cell metabolism [78]. A detailed account of the interaction of cantharidin analogues with PP1 and PP2A is given in Galvis et al. [78].

**Figure 3:**
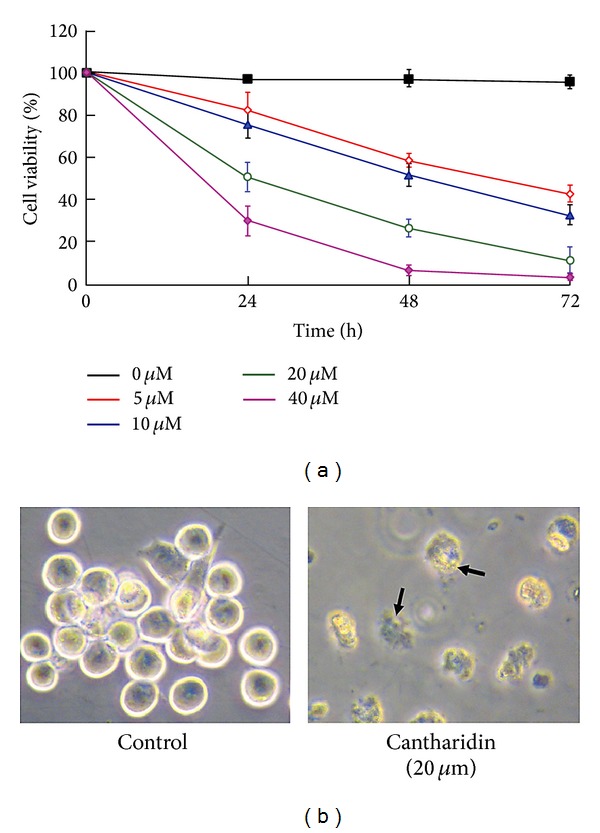
Effects of cantharidin on cell viability and morphological changes of human colorectal cancer cells. (a) Cells treated with 0, 5, 10, 20, or 40 *μ*M cantharidin for 0, 24, 48, and 72 h and then harvested for determination of cell viability. (b) Cells exposed to 20 *μ*M cantharidin for 24 h and then examined for morphological changes under phase-contrast microscopy. Data represent the mean ± SD of three experiments. From Huang et al., “Cantharidin induces G2/M phase arrest and apoptosis in human colorectal cancer colo 205 cells through inhibition of CDK1 activity and caspase-dependent signaling pathways,”* International Journal of Oncology*, volume 38, pp. 1067-1073, 2011. Reprinted with permission of Spandidos Publications 2013.

Recently, a number of papers have been published showing that cantharidin, apart from inhibiting PP1 and PP2A, has multiple effects on cancer cells. Huang et al. [85] showed that growth inhibition and killing of human colorectal cancer cells by cantharidin was both time- and dose-dependent (Figure 3). The cantharidin exposure reduced CDK1 kinase activity which led to failure of the cells to progress from G2 to M phases in the cell cycle. In addition, the colorectal cells were killed by apoptosis which was induced through the mitochondrial and death receptor pathways and activation of caspases 8, 9, and 3 (Table 4).

Another study by Huang et al. [86] on metastasis of human bladder carcinoma cells, showed that exposure to cantharidin blocked the gene expression, protein levels, and activities of the matrix metalloproteinase-2 (MMP-2) and/or MMP-9. These enzymes are associated with invasive properties of many cancers so that cantharidin had an antimetastatic effect possibly by targeting the p38 and JNK1/2 MAPKs pathway of the bladder cancer cells. Other effects of cantharidin have been studied in human breast cancer cells by Shou et al. [87]. They reported that cantharidin resulted in apoptosis and reduced growth, adhesion, and migration of the cancer cells. The reduced adhesion resulted from repression of cell adhesion to platelets through downregulation of the *α*2 integrin adhesion molecule on the surface of the cancer cells. The repression of the *α*2 integrin occurred through the protein kinase C pathway probably due to PP2A inhibition (Table 4).

Three further studies indicate novel approaches in the use of cantharidin. Lissina et al., [79] in a chemical-genomics study, showed that cantharidin is an effective gene probe of transcriptional regulation of the CRG1 gene, an uncharacterised methyltransferase, during cantharidin stress. Therefore by using such small molecules the authors showed how it was possible to elucidate unknown mechanisms of therapeutic action in cells involving, for example, the methyltransferase. Li et al. [88] have used the knowledge of the inhibition of PP1 and PP2A by cantharidin, and the resulting apoptosis of cancer cells, to design a new gene therapy approach to kill hepatocellular carcinoma cells. They inhibited PP2A using the *α*-fetoprotein promoter enhancer linked to the pgk promoter to drive the dominant negative form of the PP2A catalytic subunit. Finally, and most important for therapeutic use of cantharidin, Dang and Zhu [89] have tackled the problems of toxicity, insolubility, and short half-life in circulation of this drug by designing cantharidin solid lipid nanoparticles as drug carriers which can be given orally (Table 4).

## 6. Antimicrobial Peptides (AMPs)

The dried bodies and secretions of insects have been widely used in Folk Medicine to treat numerous diseases and illnesses including many different types of infections and cancers [1–3]. In Chinese Medicine, numerous species of insects have been used to treat cancer [2]. Considering that many insects thrive in inhospitable environments teeming with microorganisms, such as dung or rotting corpses, it is not surprising that they have robust immune defences to counter infection. These insect innate immune defences have both cellular and humoral components [90, 91], but it is the humoral antimicrobial peptides (AMPs) that are of most interest for the development of new antibiotic drugs.

Insect AMPs have been actively researched for over 50 years and in 2011 work involving these molecules led to the Nobel Prize for Physiology and Medicine being awarded to Jules Hoffmann and Bruce A. Beutler for their discovery of the Toll receptors and mechanisms of activation of innate immunity. Their work did much to increase interest in AMPs which have recently been the subject of extensive reviews [92–96]. This interest has also been fuelled by the urgent need to combat the ever increasing number of antibiotic-resistant pathogens such as MRSA, TB, and gonorrhoea. Despite this urgency, and the length of time AMPs have been studied, very few of these molecules have undergone clinical trials or, those that have, failed to complete the trials [97]. There are many reasons for the slow development of AMPs into new therapeutic drugs and these are discussed in detail below together with recent progress in this area.

### 6.1. Basic Characteristics of Insect AMPs

The LAMP 2013 database, links information on AMPs and holds 5547 AMP sequences of which 3904 are natural AMPs, while the other 1643 are synthetic peptides [97]. Interestingly, of the 5547 AMPs, 5362 have antibacterial activity, 1616 antiviral, 1579 antifungal, 138 antitumour, and 14 antiparasitic activities. The amino acids composing these AMPs range from 4 to 99 in number [97]. Insect natural AMPs previously identified are estimated as 400–500 in number [3]. AMPs are produced by bacteria, fungi, numerous invertebrates, vertebrates and plants, and are usually associated with killing microbes although they may also be involved in wound repair, inflammation, development, chemotaxis, and cytokine activity (e.g., [95, 96, 98]).

Insect AMPs are mainly cationic (although anionic forms do exist) which facilitates their binding electrostatically to negatively charged bacteria and tumour cell surfaces, whilst neutrally charged normal cells are unaffected [3]. They are also amphipathic in their folded state with hydrophilic and hydrophobic regions mediating their solubility in phospholipid cell membranes. These interactions of the AMPs result in their membrane disruptive properties which characterise these molecules [99]. Most of the insect AMPs are freely circulating or associated with the gut or other epithelia and often placed strategically at external openings on the body to combat infection [3]. Some AMPs are constitutively expressed but the majority is rapidly induced following exposure to would-be invaders. Any one insect can produce multiple AMPs which enable it to differentiate between invading organisms and to respond selectively. Many of the venom proteins such as melittin, described in Section 3.2, above, are also polypeptides with amphipathic and cationic properties but are highly toxic and confined to venom sacs to combat other predatory insects and animals.

Insect AMPs can be classified into 3 groups [3], although 4 or 5 groups have also been recognised [92, 93].

*(1) Linear *α*-helical AMPs,* which in insects include the cecropins, moricin, sarcotoxin, and melittin, are present in a wide range of insect orders, including coleopterans, dipterans, and lepidopterans. Cecropins are active against Gram-positive and Gram-negative bacteria, viruses, protozoans, fungi, nematodes, and tumour cells [3, 100]. Cecropins are promising anticancer drugs when combined with melittin (see Section 3.2) or with chemotherapy agents to reduce their toxic side effects [101]. Also, overexpressed defensin A and cecropin A genes in transgenic* Aedes aegypti* blocked the transmission of* Plasmodium gallinaceum* [102]. Recently, the potential development of engineered cecropin A-melittin analogues and other AMPs as drugs against protozoan parasites such as* Leishmania* has been reviewed [103].

*(2) Linear proline or glycine-rich AMPs* include drosocin, apidaecin, formaecin, and pyrrhocoricin. These are short, proline-rich with specific intracellular targets in bacteria, while mammalian cells are unaffected [3]. They generally target Gram-negative bacteria such as* Escherichia coli* and kill over several hours, in contrast to the other two groups of AMPs which kill rapidly. The bacterial target of the proline-rich AMPs is believed to be the intracellular chaperone DnaK [104]. Ostorhazi et al. [104] synthesised a proline-rich, designer peptide, A3-APO, and showed its efficacy against multidrug resistant bacterial infections in the wounds and lungs of mice. A3-APO upregulated the expression of the antiinflammatory cytokines interleukin-4 and interleukin-10 so that wounds lacked pus [104]. A shortened version of apidaecin, Api88, has similar activity to A3-APO against pathogenic* E. coli* but is unstable in serum. Simply substituting Arg-17 with l-ornithine increased, by more than 20-fold, the serum stability of Api88 [105].

*(3) Cysteine-stabilised AMPs* are small cationic peptides with 33–46 amino acids, and are stabilised by cysteine residues forming disulphide bridges [3]. They are common in most insects and include defensins or defensin-like compounds such as gallerimycin, heliomycin, sapecins, drosomycin, spodoptericin, and phormicins [106]. They are mainly active against Gram-positive bacteria and fungi but are also antiparasitic [103]. The defensins, like the cecropins and analogues of pyrrhocoricin, have considerable potential for development as drugs since short synthetic forms of insect defensins inhibit MRSA and disrupt myeloma cancer cells [3]. Furthermore, in mammals, defensins have the dual activities of killing bacteria as well as modulating the immune response by recruiting and activating immune cells [107]. Invertebrate defensins exhibit high affinity binding to the bacterial cell wall precursor, lipid II, and inhibit its incorporation into the peptidoglycan network [107].

### 6.2. Killing Mechanisms of AMPs

This is considered briefly as it is relevant to understanding the therapeutic use of AMPs. The cationic AMPs are amphipathic with a net positive charge with large numbers of cationic amino acids such as arginine, histidine, and/or lysine and also contain hydrophobic residues [95]. The prevalent negative net charge of bacterial membranes due to the composition of their phospholipids (predominantly with negative charge) plays a major role in the attraction of the cationic AMPs, while membranes of eukaryotic cells enriched in zwitterionic phospholipids and cholesterol are refractory to the AMPs. The cationic AMPs bind to the anionic residues of the outer bacterial envelope, which include the lipopolysaccharides of Gram-negative bacteria and the lipoteichoic acids of Gram-positive forms [93]. Binding to the outer bacterial cell membrane does not, in contrast to antibiotics, involve specific receptors for the AMPs so that it is more difficult for the bacteria to mutate and evolve resistance to the AMPs [93]. This binding results in disruption and permabilisation of the outer bacterial cell membrane and eventually microbial death.

There are a number of models for the mechanisms involved in bacterial membrane disruption. These include the barrel stave, the carpet, the toroidal, and ion channel forming models. These models have been extensively reviewed previously (e.g., [92, 99, 108, 109]). In the barrel stave model, clusters of *α*-helical AMPs are inserted in the membrane like a barrel with the staves (strips of wood forming the wall) forming a transmembrane pore. The hydrophilic side groups of the AMPs line the aqueous pore, while the hydrophobic tails of the phospholipid membrane fatty acids interact with the nonpolar side groups of the AMP. In the carpet model, the AMPs attach parallel with the membrane, form a carpet resulting in holes in the membrane which then, at a critical concentration, collapses. With the toroidal model, the AMPs are inserted perpendicular into the membrane to form pores lined by the AMPs and the lipid head groups. Finally, with the ion channel forming model, the AMPs bind to the polar head groups, insert into the membrane, aggregate, and span the membrane to form pores through which ions leak from the bacterial cell [93]. The same membanolytic activities of AMPs would apply to their killing of anionic cancer cells [100].

Evidence, however, is accumulating that the activity of AMPs is probably not confined to cell membrane lysis. Thus, AMPs may disrupt mitochondrial membranes, inhibit cell wall synthesis, inhibit DNA synthesis, inhibit protein synthesis, interact with membrane receptors and heat-shock proteins, and have antiangiogenesis effects [92, 99, 100]. One example is apidaecin which kills Gram-negative bacteria without forming pores and interferes with protein synthesis [99].

### 6.3. Therapeutic Use of AMPs

AMPs have great potential for development as new classes of antibiotics for a number of reasons.

- There is a huge variety, targeting a broad range of microorganism and cancer cells and lending themselves to synthetic improvement.
- They seem to have multiple targets and do not generally rely on specific receptor binding so that development of resistance by bacteria is difficult.
- They generally kill rapidly and within minutes while conventional antibiotics usually take hours.
- They can kill antibiotic-resistant bacteria such as MRSA as well as cancer cells.
- Their antimicrobial activity occurs even with low micromolar, concentrations.
- They may have dual effects to kill microbes and to modulate the immune system.
- They can destroy biofilms on medical devices even when used at low concentrations.

Despite the advantages of AMPs, progress to date in developing them for clinical use has been disappointing. The main advance has been with vertebrate AMPs for use in topical applications and a few AMPs have entered clinical trials [3, 92, 108, 110]. These AMPs were designed for a number of external uses such as skin care, acne, eye infections, and catheter-related pathogens.

There have been a number of reasons for this slow progress in AMPs becoming available for clinical use.

(1) Lack of interest by large pharmaceutical companies for many years. Thus, between 1998 and 2004, of the 290 new antibacterial drugs under development, only 4 involved the major pharmaceutical companies [3]. This attitude is now changing for various reasons including the emergence, in the last 10 years, of more and more antibiotic-resistant bacteria and fewer and fewer antibiotics available to treat these pathogens. In addition, research has revealed the dual function of some of these AMPs with the ability not only to kill microbes but also to modulate the immune system [111]. No doubt this fact has not escaped the attention of the pharmaceutical companies with the potential of developing new classes of drugs able to control immune reactivity. In addition, much progress has also been made in understanding the functioning of the AMPs which can be produced at much lower costs than their natural counterparts [95]. The more effective delivery of AMPs by gene therapy or nanoparticles is also being developed and will enhance the therapeutic potential of AMPs ([50, 51], see Section 3.2) and again raise interest in this rapidly evolving subject.

(2)  High production costs have always been a major hurdle to development of the AMPs since they only occur at low concentrations naturally and the cost of solid phase synthesis is very high [3]. Advances, however, are rapidly being made to reduce manufacturing costs. Thus, numerous reports describe the synthesis of truncated synthetic analogues with enhanced killing activities and with potential for cheaper production costs. For example, Ausbacher et al. [112] designed a series of small antimicrobial *β* ^2,2^-amino acid derivatives of Mw < 500, with potent activity against both MRSA and cancer cells, and Gaspar et al. [100] also described a number of short, synthetic peptides for use against different types of solid tumours. These latter peptides included four enantiomeric AMP analogues (D-peptides A, B, C, and D) designed from beetle defensins [113]. An alternative strategy to reduce costs of mass production is to use recombinant technology but this has been hindered by the antibacterial activity of the AMPs and their proteolytic degradation during production [114]. Recent studies, however, have used cost effective, modified recombinant techniques with* E.coli* or with the methylotrophic yeast* Pichia pastoris* as vectors, to produce fully functional insect cecropins capable of killing a range of bacteria, including MRSA [115, 116]. Finally, regulatory rules governing the required performance prior to approval for the release of drugs in the USA (FDA) and Europe have all to be navigated at extra cost [3]. It is noteworthy that much of the significant research on AMPs is now being conducted in China.

(3) There are also concerns about the stability and toxicity of the AMPs towards mammalian cells [93, 94] as well as the development of bacterial resistance to these molecules. However, as a result of better understanding of the structural-functional relationships of AMPs and the introduction of computer modelling, it is now possible to design and synthesise AMPs with increased stability in serum and saline, no toxicity, and greater killing activities [3, 117]. These AMPs have been produced by amino acid substitutions, sequence splicing, and changes in ratios of hydrophobic amino acids to produce truncated designer compounds against clinically isolated, antibiotic resistant pathogens at low MICs of <10 *μ*g/mL [117]. Further advances will occur in isolating and synthesising active AMPs with the introduction of new discovery pipelines utilising in silico designed AMP-encoding oligonucleotide libraries [118] and advanced quantitative structure-activity relationship (QSAR) models [119].

Regarding development of resistance of bacteria to AMPs, this was thought to be less likely to occur than with conventional antibiotics as AMPs may have multiple sites of action within the bacterial cell and involve fundamental changes in the membrane (e.g., [120]). This view, however, has been shown to be over optimistic as reports have appeared of resistance to AMPs [3], including resistance to insect melittin and cecropin [121, 122]. In addition, the evolution of resistance to a cationic AMP has been shown through continual selection in the laboratory [123] although conditions in nature are very different. Thus, each host will contain a different range of AMPs in the various tissues of the body. This point is emphasised by Chernysh and Gordja [124] who prepared a* Calliphora vicina *maggot peptide complex called FLIP7 (from Fly Larvae Immune Peptides) containing cecropins, defensins, diptericins, and a proline-rich peptide and compared its ability with the antibiotic, meropenem, to kill a multiresistant strain of* Klebsiella pneumonia* over many generations. The results showed that after 25 passages with the meropenem, the resistance of the bacteria was increased 128 times, while there was no change in resistance towards the* C. vicinia *FLIP7 complex. Thus, although resistance to AMPs can occur, this should not deter their development as therapeutics but their widespread use should be carefully regulated [123]. The use of hybrid molecules constructed, for example, from cecropin and melittin or cecropin and rifampicin should also help to solve this problem [125].

In conclusion, the future of the development of AMPs as new classes of drugs for killing antibiotic resistant bacteria and cancer cells looks very bright. The versatility of these potential drugs seems to increase daily with, for example, recent reports of the use of AMPs to coat titanium bone implants to prevent infection [126] and the inhibition of biofilm formation by these compounds [125].

## 7. Maggot Molecules

**Figure 4:**
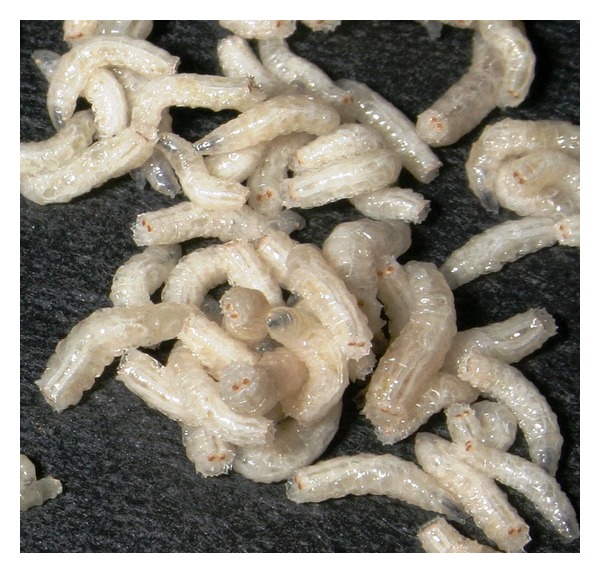
Showing newly washed final instar* Lucilia sericata* larvae prior to incubation for production of extracorporeal secretions. Photograph by kind permission of Mr. I.F. Tew, Swansea University.

Maggots have been used for wound healing in Folk Medicine by the aborigines and Mayan Indians for thousands of years. Maggots for cleaning wounds also occurred in the Napoleonic and American Civil Wars [3, 127]. However, maggot therapy only obtained wider recognition for treating infected wounds after its introduction into USA hospitals in the 1920s by Professor William Baer at John Hopkins University. By the 1930s and 1940s over 300 USA hospitals used this procedure and it had also spread to Europe [3, 127]. In the 1940s, however, the newly discovered antibiotics soon dampened the enthusiasm for maggot therapy and only the appearance of antibiotic-resistant bacteria in the 1980s rekindled interest in this procedure. Maggot therapy is now commonly used for many types of infected wounds such as diabetic foot wounds, postoperative infections, bed sores, and leg ulcers, in the USA, Israel, and Europe [3, 127]. It is estimated to have saved the NHS, UK over 500 million pounds. The larvae of the blowfly,* Lucilia sericata, *are frequently used (Figure 4) although other species have also been tried such as* Lucilia cuprina, Phormia regina, and Calliphora vicina* [127]. Thus, a wide spectrum of dipteran species has potential as sources of new medicinal drugs, especially since the larval stage of* L. sericata* can kill MRSA [128]. Recent reviews of maggot therapy provide more details of the processes involved [129–131].

The use of* L. sericata* larvae for treating wounds has been recognised by the U.S. Food and Drug Administration and the UK Prescription Pricing Authority. Sterile maggots can therefore be officially prescribed (http://www.medicaledu.com/maggots.htm).

Maggot therapy can be divided into 3 processes:

- debridement of wounds;
- wound healing;
- disinfection of wounds.

### 7.1. Debridement of Wounds

Once maggots are applied to the wound then debridement or cleaning and removal of necrotic tissue and debris (eschar) occur so that granulation and healing can begin. Maggots clean wounds by the extracorporeal production of enzymes (Table 5) that digest the debris which the maggots then feed upon [3]. Initially, the main enzymes identified in the maggot excretions/secretion (ES) were chymotrypsin- and trypsin-like serine proteases, an aspartyl proteinase and a metalloproteinase [132]. The secretion of ammonia by the maggots increases the pH to activate the serine proteases. The most active enzymes are produced by first instar larvae [132].

More recent work from the Pritchard group in Nottingham University has revealed more information about the maggot proteases and also detected other enzymes present in the MS. First; the* L. sericata* chymotrypsin I is resistant to the endogenous wound protease inhibitors, *α*1-antichymotrypsin and *α*1-antitrypsin, present in eschar and which could potentially inhibit debridement [146]. In contrast, mammalian *α*-chymotrypsin is inhibited by these enzymes so that maggot chymotrypsin I can survive in the wound to undertake debridement, whilst the mammalian enzyme cannot. Second, the MS have also been shown to contain a DNase able to degrade genomic bacterial DNA, the extracellular bacterial DNA in preformed biofilms from a* Pseudomonas aeruginosa* clinical isolate, and DNA from the slough/eschar of a venous leg ulcer [133]. This DNase must make a valuable contribution to debridement and healing by clearing tissue DNA as well as that of biofilms, thus freeing tissue protein for digestion by the ES proteases [133]. Third, the ES also contain glycosidases which would remove sugars from the wound debris and contribute to the debridement process [134]. All these enzymes (Table 5) in the ES remove the extracellular matrix debris, fibrin clots, and any biofilms associated with infecting bacteria and allow healing to begin [147–150]. All the above work was undertaken in* L. sericata* but recent work investigating the potential of another calliphorid species,* Sarconesiopsis magellanica*, for use in maggot therapy has shown that larval ES of this insect also contain trypsin-like serine proteases [151].

Pritchard and colleagues have applied their findings to develop hydrogel bandages containing these enzymes in order to accelerate debridement and healing processes [152]. Recently, they have made additional progress by producing a recombinant chymotrypsin I, using good medical practice guidelines, which successfully digested wound debris and is now available for clinical trials [153, 154].

### 7.2. Wound Healing

There is no doubt about the benefits of maggots in debriding chronic wounds but the outcome of clinical trials on their use in wound healing is more uncertain [155]. The ES enzymes or other constituents have been shown to activate the fibroblasts [156] and evidence is accumulating for an active role for ES in wound healing. Thus, specific amino acids derivatives and fatty acids extracts (Table 5) from* L. sericata* ES induce mitosis in human endothelial cells and activate angiogenesis and wound healing [135, 136].

In addition, there is accumulating evidence that ES have an immunomodulatory role in the wound healing process (Table 5) and this has been reviewed in detail previously [131]. In particular, neutrophils, macrophages, lymphocytes, and the complement system respond to exposure to the MS. With neutrophils, the ES inhibit elastase, the respiratory burst, hydrogen peroxide production, and migration of these cells. Elastase breaks down the extracellular matrix and delays epithelial repair, while oxygen radicals would probably have a similar effect. Concomitantly, the inhibition of neutrophil migration would help resolve the prolonged inflammatory response, to which they contribute, present in a chronic wound [131, 137]. Macrophages are similarly affected by the ES and show reduced migration and inhibited production of proinflammatory cytokines such as migration inhibitory factor and TNF-*α*. At the same time, the production of the anti-inflammatory cytokine IL-10 is increased so that the ES appear to be reducing the inflammatory response [138]. In addition, in the presence of ES, macrophages develop into anti-inflammatory rather than proinflammatory forms [139]. The anti-inflammatory macrophages suppress inflammation and secrete basic fibroblast growth factor (bFGF) and vascular endothelial growth factor (VEGF) which mediate mitosis and migration of endothelial cells resulting in angiogenesis and eventual healing of wounds [157]. Recently, these results were confirmed by applying ES to acute skin wounds made in rats since levels of the acute inflammatory cytokines, TNF-*α* and IL-6, remained significantly lower than in the rats with untreated wounds [157]. Lymphocytes activation too is inhibited by the ES so that the wound site would be protected from the induction of an adaptive response to the maggot proteins [140].

Even more interesting is the study by Cazander et al. [141] who have shown that ES could reduce complement activation by 99.99% in the sera of healthy and postoperatively immune-activated human patients. The ES break down complement components C3 and C4 which could explain, in part, the improved wound healing following maggot therapy (Table 5).

### 7.3. Disinfection of Wounds

There is good evidence that ES can kill bacteria infecting wounds, including antibiotic-resistant strains such as MRSA [3]. There are reports of many different antibacterial factors in dipterans, including a range of AMPs such as Sarcotoxin 1A, a cecropin-like molecule from the flesh fly* Sarcophaga peregrine,* which is more active against Gram-negative bacteria than Gram-positive forms [158]. However, focus is now on calliphorid flies used in wound healing in which one AMP, lucifensin (Table 5), has received particular attention recently as it is active against clinically relevant bacteria such as* Streptococcus* species (e.g., [143]). Most of the other antibacterial factors described from calliphorids are <1300 Da in size [3], although Altincicek and Vilcinskas [149], and Andersen et al. [143] have shown that* L. sericata* has 65 immune-inducible genes including lysozyme- and transferrin-likegenes and 3 proline-rich AMPs.

Lucifensin was first purified in 2010 from an extract of the gut of* L. sericacta* larvae by Čeřovský et al. [142]. They showed that the peptide contained 40 amino acid residues and 3 disulphide bridges and was a typical 4 kDa dipteran defensin. Subsequently, Andersen et al. [143] published the primary sequence, and Čeřovský et al. [159] chemically synthesised lucifensin to provide material for a structural-activity study. More recently, lucifensin II was discovered and characterised from* Lucilia cuprina* and found to be identical to the* L. sercata* lucifensin except for one amino acid residue [160]. Thus, lucifensins are cationic AMP with main activity against Gram-positive bacteria [143] so that, together with seraticin (see below), they make an important contribution in the ES to cleaning infected wounds of MRSA and other antibiotic-resistant bacteria. This antibacterial activity occurs even at physiological salt levels [138]. Lucifensin is present in the gut, fat body, and haemolymph of* L. sericata *and appears to be constitutively expressed [142, 160]. In addition, in* L. sericata* orally challenged with bacterial isolates from wounds, only in the fat body is there an increase in lucifensin expression so that levels in the ES remain unchanged [161]. Lucifensin has also been studied in a detailed structural analysis by NMR [162]. It seems possible that it has two mechanisms of antimicrobial activity against the bacterial cell and interacts both with the bacterial membrane and binds to the cell wall precursor, lipid II [162]. Finally, interest has also increased in the antibacterial factors of the house fly,* Musca domestica*, because of its possible role as a vector of pathogens such as MRSA [163, 164]. Results show that these insects also produce a defensin that is upregulated upon bacterial ingestion and that this, and probably other factors, is responsible for the antibacterial activity against MRSA and VRE (vancomycin-resistant enterococci) recorded for solvent extracts of maggots [164].

As far as the calliphorid low weight antibacterial factors are concerned, there are two sets of molecules for development as new medicinal drugs, namely, the alloferons and seraticin (Table 5).

Two alloferons were originally isolated from the haemolymph of* Calliphora vicinia* by Chernysh and colleagues [144] and are peptides with amino acid sequences of HGVSGHGQHGVHG (alloferon 1) and GVSGHGQHGVHG (alloferon 2). Synthetic alloferon in* in vitro* tests stimulated natural killer cells, while* in vivo* interferon was induced in mice. There were also indications of antiviral and antitumour activities [144] with alloferon also showing moderate tumoristatic and tumoricidal activities in transplanted tumours in mice [165]. More recently, a derivative of alloferon, allostatin, has been shown to have a significant adjuvant effect in vaccination experiments against tumour cells in mice [166]. Clinical studies by Ryu et al. [167] subsequently showed that alloferon activates immune cells through the NF-kappaB signalling pathway. The Allopharm Company was then formed in Russia and Allomedin was marketed in 2005 for the treatment of genital herpes, cold sores, and gingivitis [3]. A number of detailed structural-functional studies have been undertaken of alloferon by sythesising analogues with amino acid substitutions in position 1, for example, in the peptide chain [7, 168]. Some of these analogues extended the antiviral properties of the native molecules so that they inhibited not only human herpes virus 1 but also coxsackievirus multiplication* in vitro.* Another study has shown the therapeutic potential of alloferon for the treatment of Kaposi's sarcoma, caused by the Kaposi's sarcoma-associated herpesvirus, and a characteristic condition in HIV patients [169].

Seraticin is present in the MS of sterile* L. sericata* larvae [128, 145]. The MS has antibacterial activity against both Gram-positive and Gram-negative bacteria including* S. aureus*, MRSA,* Bacillus thuringiensis*,* E. coli*,* P. aeruginosa,* and* Enterobacter cloacae*. The fact that maggot samples collected with the highest pH also had the highest antibacterial activity probably eliminates phenylacetic acid, produced by the commensal,* Proteus mirabilis,* as the source of the factor involved [145, 170]. Subsequently, further fractionation of maggot secretions revealed a fraction of <500 Da active against* S. aureus*, 10 strains of MRSA, and a number of Gram-positive and Gram-negative bacteria [128]. This <500 Da fraction, named “seraticin,” has been the subject of additional research, due to its inhibition and killing of clinical strains of MRSA and* Clostridium difficile,* and has been isolated and characterised, and an empirical formula was calculated. Mass spectrometry and NMR studies have been carried out and a synthesis has produced fractions having similar antimicrobial properties to the native seraticin molecule. A <1000 Da molecule, active against MRSA, and from sterile* L. sericata* larvae, has also been reported previously [171]. Unfortunately, lack of funding seriously delays the development of research and commercialization of such interesting and potentially important new medicines, especially if derived from such unfashionable sources as fly maggots.

## 8. Insect Anticoagulants

The anticoagulants in the salivary glands of blood sucking ticks and insects such as the Hemiptera, Diptera, Siphonaptera, and Anoplura have tremendous potential for development of new anticoagulants and immune modulating medicines [3]. In fact, extracts of the salivary glands of horseflies have been used for centuries in Eastern Medicine as anticlotting agents [3]. Progress, however, has been made in identifying and commercialisation of such invertebrate anticoagulants not from insects but from leeches and ticks [1, 3]. In leeches, recombinant derivatives of hirudin have been made available commercially for some years in Europe and the USA [172] with the approval of the FDA. With ticks, intense research is underway on the bioactive substances produced by their salivary glands and a variety of molecules with diverse functions have been described with potential use as pharmaceuticals (reviewed in [173]). Attention has been focused on ticks probably due to the variety of pathogens vectored by these animals [173].

**Figure 5:**
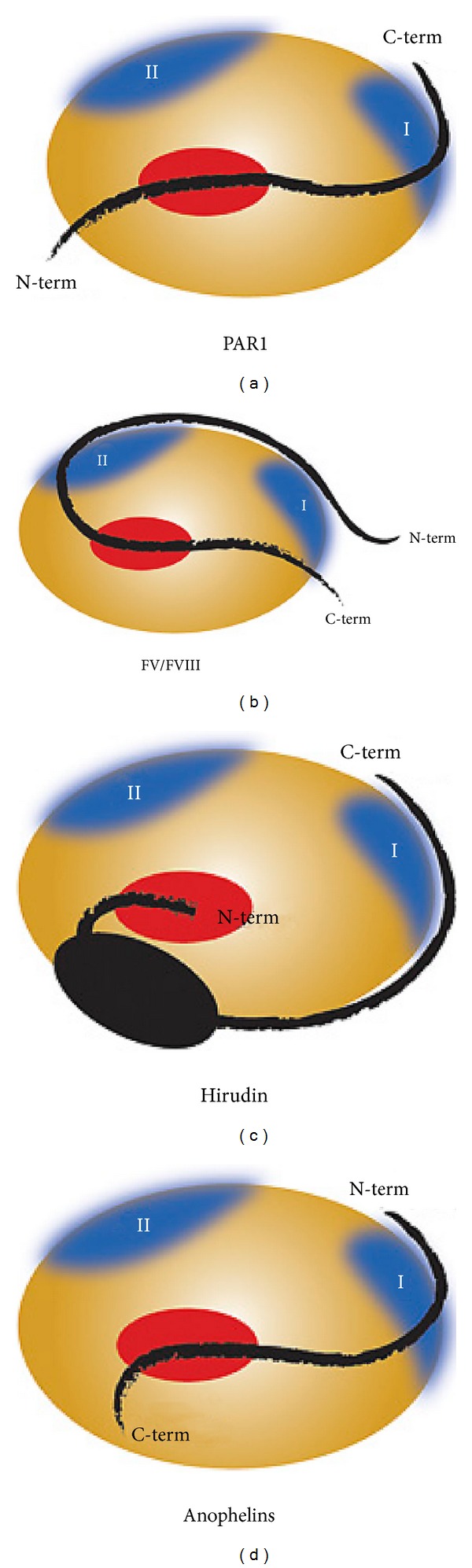
Comparison of mechanism of thrombin recognition by anophelin with other substrates and inhibitors. Thrombin is represented by an orange ellipse, with exosites in blue and the active site in red. Anophelin inhibitor binds to thrombin in a reverse orientation relative to the other molecules, such that the N-terminal portion recognises exosite I, whereas the C-terminal acidic segment binds to the active site of thrombin. From original Figure 4 of Ana C. Figueiredo, Daniele de Sanctis, Ricardo Gutiérrez-Gallego, Tatiana B. Cereija, Sandra Macedo-Ribeiro, Pablo Fuentes-Prior, and Pedro José Barbosa Pereira, “Unique thrombin inhibition mechanism by anophelin, an anticoagulant from the malaria vector” which appeared in Proceedings of the National Academy of Sciences of the United States of America.” Volume, 109, Issue 52, pages E3649 to E3658, 2013 (doi: 10.1073/pnas.1211614109). Reprinted with permission of PNAS.

Regarding insects, much less is known about the anticoagulants in their salivary glands possibly due to the sheer numbers of protein families produced in these glands [174]. However, a recent thorough analysis of the structure and function of thrombin inhibition by anophelin in the salivary glands of* Anopheles* mosquitoes has been made and discovered a unique thrombin inhibition mechanism [175]. Thrombin is an atypical (chymo) trypsin-like enzyme, with a narrow active site cleft for specific substrate recognition and also has secondary recognition surfaces (exosites) [175] In contrast to other natural bivalent inhibitors of thrombin which bind to one of the thrombin exosites through their C-terminals, anophelin shows reverse binding to an exosite by means of the N-terminal and the C-terminal binds to the active site as shown in Figure 5 [175]. The significance of this finding is that it imparts anophelin with potent inhibitory properties as well as high resistance to proteolysis by thrombin and this may have implications for the design of novel antithrombotics [175].

## Figures and Tables

**Table 1:** Examples of phenols present in honey with anticancer properties*.

Class of phenolic compounds	Examples of specific phenolic compounds researched
(1) Flavonols	Quercetin, kaempferol, galangin, fisetin, and myricetin
(2) Flavanones	Hesperidin
(3) Flavones	Apigenin, acacetin, chrysin, luteolin genkwanin, wogonin, and tricetin
(4) Phenolic acids	Caffeic acid
(6) Coumarins	Coumarin
(7) Tannins	Ellagic acid

*Table modified from Abubakar et al. [20].

**Table 2:** Examples of strategies to overcome the cytolytic properties of melittin.

Strategy	Target	References
(1) Cancer cells killed by dilutions of melittin not affecting normal cells	Lung cancer cells *in vitro *	Zhu et al. [45]
(2) Point mutation and deletion of specific melittin amino acids	Reduced haemolysis of normal cells but inhibition of bacteria	Zhao et al. [46]
(3) Synthetic melittin coupled to hecate-CGb^a^ as a delivery vehicle	Ovarian, testicular, and adrenocortical tumours *in vivo *	Vuorenoja et al. [47]
(4) Melittin coupled to a specific homing peptide identified by phage display	Hepatocellular carcinoma cells* in vitro *	Zhao et al. [48]
(5) Gene therapy and transfection of melittin gene into tumours	Human bladder carcinoma cells* in vitro *	Winder et al. [49]
(6) Use of nanoparticle technology for delivery of melittin to tumours	Melanomas *in vivo *	Soman et al. [50] Huang et al. [51]

^a^Hecate-CGb: the beta chain of human chorionic gonadotropin.

**Table 3:** Examples of potential use of silk biopolymers in medicine.

Form of Silk	Potential Use	References
(1) Nanoparticles	Delivery of drugs to cancer cells	Numata and Kaplan [59] and Nitta and Numata [62]
(2) Co-polymer blocks	Transfection of target cancer cells	Numata et al. [58] and Numata and Kaplan [59]
(3) Small, globular units with tumour homing peptides (THP)	Improved tumour cell-specific transfection	Numata et al. [66]
(4) Nano-scale silk-based ionic complexes with THP	Further improved tumour cell-specific transfection	Numata et al. [67]
(5) *B. mori* porous materials	For repair of cartilage, bone, ligaments, tendons, vascular tissue, nerves, corneas and as wound dressings	Zhang et al. [68]
(6) Silk-heparin support	Vascular tissue growth application	Seib et al. [69]
(7) Silk hydrogels	Treatment of breast cancer	Seib et al. [70]
(8) Antibiotic-loaded silk hydrogels	Prevention and treatment of infection	Pritchard et al. [71]
(9) Electrically stimulated silk films	Enhancement of neural growth	Hronik-Tupaj et al. [72]
(10) Silk protein matrices	Thermostabilisation of vaccines	Zhang et al. [73]
(11) Vitamin-E loaded silk nanofibrous mats	Skin tissue regeneration	Sheng et al. [74]

**Table 4:** Examples of recent studies on the use of cantharidin.

Cells treated	Results	References
(1) Human colorectal cancer cells	Reduced CDK1 kinase activity, apoptosis induction through mitochondrial and death receptor pathways, and activation of caspases 8, 9, and 3	Huang et al. [85]
(2) Human bladder carcinoma cells	Blocked activities of matrix metalloproteinase-2 (MMP-2) and/or MMP-9 resulting in an antimetastatic effect	Huang et al. [86]
(3) Human breast cancer cells	Reduced adhesion and migration by repressed cell adhesion to platelets by downregulation of *α*2 integrin adhesion molecule	Shou et al. [87]
(4) Yeast CRG1 (cantharidin resistance gene 1)	Details of transcriptional regulation of the CRG1 gene for methyltransferase, during cantharidin stress	Lissina et al. [79]
(5) Hepatocellular carcinoma cells	A new gene therapy approach to kill hepatocellular carcinoma cells by inhibiting PP2A with the *α*-fetoprotein promoter enhancer linked to the pgk promoter	Li et al. [88]
(6) Normal rats	Design of cantharidin solid lipid nanoparticles as drug carriers which can be given orally	Dang and Zhu [89]

**Table 5:** Summary of factors/processes involved in maggot therapy of infected wounds.

Factors/processes mediated by maggot extracts and secretions	Effect on wound	References
*Debridement *		
(1) Maggot proteases	Digest wound debris	Chambers et al. [132]
(2) Maggot DNase	Digest DNA of debris and infecting bacteria in biofilms	Brown et al. [133]
(3) Maggot glycosidases	Digest wound debris	Telford et al. [134]
*Wound healing *		
(4) Specific amino acids	Induce mitosis in endothelial cells	Bexfield et al. [135]
(5) Maggot fatty acid extracts	Activate angiogenesis	Zhang et al. [136]
(6) Neutrophil migration inhibition	Resolves inflammation	van der Plas et al. [137]
(7) Macrophage migration inhibition and TNF-*α*.	Resolves inflammation helped by increased IL-10	van der Plas et al. [138]
(8) Anti-inflammatory macrophages increased	Resolves inflammation helped by bFGF and VEGF cytokines inducing mitosis and angiogenesis	van der Plas et al. [139]
(9) Lymphocyte activation suppressed	Inhibits adaptive immunity to maggot proteins	Elkington et al. [140]
(10) Reduced complement activation	Inhibits complement action against maggot proteins	Cazander et al. [141]
*Wound disinfection *		
(11) Maggot lucifensin	Active against gram-positive bacteria, for example, MRSA	Čeřovský et al. [142] Andersen et al. [143]
(12) Maggot alloferons	Antiviral and antitumour activities	Chernysh et al. [144]
(13) Maggot seraticin	Active against gram-positive and gram-negative bacteria	Bexfield et al. [145]

## Final Comment

The authors apologise to those scientists who have provided invaluable information for this review but whose publications have only been cited indirectly via relevant reviews.
